# The Readability of Electronic Cigarette Health Information and Advice: A Quantitative Analysis of Web-Based Information

**DOI:** 10.2196/publichealth.6687

**Published:** 2017-01-06

**Authors:** Albert Park, Shu-Hong Zhu, Mike Conway

**Affiliations:** ^1^ Department of Biomedical Informatics University of Utah Salt Lake City, UT United States; ^2^ Department of Family Medicine & Public Health University of California, San Diego La Jolla, CA United States

**Keywords:** electronic cigarettes, tobacco use cessation products, health services, consumer health information, health education

## Abstract

**Background:**

The popularity and use of electronic cigarettes (e-cigarettes) has increased across all demographic groups in recent years. However, little is currently known about the readability of health information and advice aimed at the general public regarding the use of e-cigarettes.

**Objective:**

The objective of our study was to examine the readability of publicly available health information as well as advice on e-cigarettes. We compared information and advice available from US government agencies, nongovernment organizations, English speaking government agencies outside the United States, and for-profit entities.

**Methods:**

A systematic search for health information and advice on e-cigarettes was conducted using search engines. We manually verified search results and converted to plain text for analysis. We then assessed readability of the collected documents using 4 readability metrics followed by pairwise comparisons of groups with adjustment for multiple comparisons.

**Results:**

A total of 54 documents were collected for this study. All 4 readability metrics indicate that all information and advice on e-cigarette use is written at a level higher than that recommended for the general public by National Institutes of Health (NIH) communication guidelines. However, health information and advice written by for-profit entities, many of which were promoting e-cigarettes, were significantly easier to read.

**Conclusions:**

A substantial proportion of potential and current e-cigarette users are likely to have difficulty in fully comprehending Web-based health information regarding e-cigarettes, potentially hindering effective health-seeking behaviors. To comply with NIH communication guidelines, government entities and nongovernment organizations would benefit from improving the readability of e-cigarettes information and advice.

## Introduction

The popularity and use of electronic cigarettes (e-cigarettes) has rapidly increased across all demographic groups in recent years [1]. In fact, there is a continuing increase in not only Web-based promotional messages for e-cigarette brands and flavors [2], but also the use of e-cigarettes by non or former-smokers[1] and youth [3]. Despite inconclusive and contested evidence regarding their safety and effectiveness in helping smoking cessation [4,5], many e-cigarette users believe that they have better health, including improved breathing, less coughing, and lesser chance of getting a sore throat when compared with combustible cigarette users [3]. Thus, analyzing readability (ie, how difficult a text is to understand) of easily accessible e-cigarette related health information and advice (EHIA) is a much needed step toward understanding available EHIA and identifying opportunities to enhance health advice practices for specific target populations.

The Internet has become a prominent source of text-based health information for consumers [6]. Meanwhile, health information is only productive if it is understood by its audience. The average American adults’ reading level is estimated to be at the 8th grade [7]. Thus, the US Department of Health and Human Services (HHS) [8] and the National Institutes of Health (NIH) [9] recommend health information to be written at 6th to 7th grade level, which is the expected reading level for age 10 to 13 years in the US education system. These recommendations are made to ensure the understandability of health information and reduce health information deficits in the general population.

A number of studies have investigated the readability of health-related content on the Internet. Across these studies, researchers consistently found empirical evidence that text-based consumer health information resources were too complex for the recommended 6th to 7th grade reading level [8,9]. For instance, smoking education materials [10], warnings on alcohol and tobacco products [11], Web-based patient education materials [12-14], informed consent documents used in clinical trial research [15], government endorsed written action-plan handouts [16], and commercially available health information [17] were found to require higher literacy levels than that recommended by the NIH and HHS. Moreover, health information available from commercially funded sources was significantly more difficult to read than information available from government-funded sources [18]. This complexity often led to comprehension errors [19,20] for average Americans. We believe that this study is the first study that examines the readability of EHIA available on the Internet.

## Methods

A systematic search of EHIA was conducted using 3 search engines (ie, Google, Yahoo, and Bing) in January of 2016. We simulated the behavior of general consumers using various combinations of search terms: *advice*, *cig*, *cigarette*, *e*, *electronic*, *health*, and *information*. Then for comparison purposes, we specifically searched for EHIA from various US public health agencies (eg, HHS), other English speaking nations’ public health agencies (United Kingdom, Australia, New Zealand, Canada), popular consumer health information sites (eg, WebMD), as well as nongovernment organizations (eg, Wikipedia).

In this study, data was only gathered from the first page of search results for each search engine, as most users rarely investigate past the first page of search results [21], and so our focus with this work is the analysis of the most frequently accessed EHIA, rather than a comprehensive study of all EHIA. We manually verified search results and retained those webpages that included any EHIA. We excluded articles published in peer-reviewed journals since general consumers are unlikely to read them. Any figures, such as pictorial descriptions, were removed and the webpages were converted to plain text for analysis.

Organization types were determined by the affiliations, funding sources, and available classification information for each organization. Several websites had no explicit indication of their affiliations or funding sources. We assumed that they were for-profit entities due to their informational advertising style content. Moreover, several documents formed part of a bigger document (eg, Wikipedia), in which case we only included sections on EHIA in this study (see Multimedia Appendix 1).

To assess readability (ie, the estimated US grade level that is required to comprehend a text), we used *Flesch-Kincaid grade level* [22], *Simple Measure Of Gobbledygook (SMOG) Index* [23], *Coleman and Liau Index* [24], and *automated readability index* [25], which are widely used metrics in previously mentioned readability studies [10-16,18]. To perform the automated readability analysis, we used the open-source Python *textstat* package [26]. In order to increase the reliability of our readability metrics, and given that different readability metrics can generate a range of results, our analysis was based on the mean of the 4 readability metrics. We then conducted pairwise independent sample *t* tests to compare readability scores among different groups (ie, for-profit entities, nongovernment organizations, non-US government entities, the US government, the US government entities written for teens) followed by *P* value adjustments using the prespecified Hommel procedure [27] to adjust for multiple comparison. The research reported in this study was exempted from review by the University of Utah Institutional Review Board (ethics committee) (IRB_00076188).

## Results

We collected a total of 54 documents for this study including materials from 27 US government entities (eg, HHS), 10 for-profit entities (eg, Consumer Affair), 7 non-US government entities (eg, Ministry of Health New Zealand), 7 nongovernment organizations (eg, Mayo Clinic), and 3 documents that were specifically written for teens by US government entities (eg, National Institute on Drug Abuse).

Complete readability scores for each document are presented in Multimedia Appendix 1. On average, the following grade reading levels (standard error) were required to understand the materials from these organizations (see Multimedia Appendix 2):

- for-profit entities: 10.46 (0.55)
- nongovernment organizations: 14.30 (0.86)
- non-US government entities: 14.44 (0.58)
- the US government: 13.48 (0.33)
- the US government entities written for teens: 10.71 (1.15)

The overall comparisons of different groups are shown in Table 1, and the details of comparison results using individual metrics are available in Multimedia Appendices 3-6. Content from for-profit entities was found to be significantly easier to read when compared with materials from all other entities except for materials written for teens by the US government. The differences among all other groups were not found to be significant (Table 1).

**Table 1:** Pairwise *t* test using average scores of 4 metrics.

Organization Type	Organization Type	*t* value	*P* value	Adjusted *P* value (Hommel)
Versus for-profit entities	Nongovernment organizations	−3.97	.001	.01
	Non-US government entities	−4.90	<.001	.002
	US government	−4.76	<.001	<.001
	US government (teen)	−0.21	.84	.89
Versus nongovernment organizations	Non-US government entities	−0.14	.84	.89
	US government	1.07	.29	.88
	US government (teen)	2.36	.05	.23
Versus non-US government entities	US government	1.36	.18	.59
	US government (teen)	3.27	.01	.07
Versus US government	US government (teen)	2.64	.01	.08

## Discussion

### Principal Findings

In this study, we used 4 different readability metrics to evaluate the readability of EHIA from 54 sources gathered on the Internet. All 4 metrics indicate that all located EHIA are written at a higher level than the recommended level for the general public. Moreover, EHIA written by for-profit entities, many of whom were advocating e-cigarettes, were significantly easier to read than materials written by nongovernment organizations, non-US government entities, and the US government. Our results contrast with the results of a previous readability study comparing health information written by commercially funded sources and government-funded sources [18]. However, both studies found that the readability of health information was generally too difficult for the public. One encouraging finding in this study is that materials written specifically for teens by US government entities were easier to read than other materials generated by US government entities aimed at the general population, although the difference was found significant for only 1 metric— *Coleman and Liau Index* (see Multimedia Appendix 5).

### Limitations

We recognize various limitations of this study. First, individuals accessing EHIA on the Web may not be representative of the general population. However, given that the Internet has become an increasingly popular resource for gathering health information in recent years [6,28], it is likely that a substantial proportion of those potential and current e-cigarettes users seeking EHIA on the Web would have experienced difficulties in fully comprehending “official” health advice, potentially hindering effective health-seeking behaviors. Second, we acknowledge that readability measures alone may not be a perfect representation of reading level [29]. For instance, EHIA could contain pictorial information, which has been shown to be more effective than text-only messages in conveying health warnings on tobacco packages [30]. In this study, we focused on textual information as text remains the primary medium for health communication and information dissemination on the Internet [31]. Third, we used general purpose readability metrics that measure rudimentary lexical features of text. Although these metrics may not be able to accurately assess the complexity of a text [32], a recent study shows lexical features are more important in estimating readability than the complexity of sentences [33]. Finally, our analysis, although systematic, is not exhaustive. A large number of EHIA exist that were not included in our study. Moreover, we limited our search to English language materials. However, we evaluated materials from key official websites that are easily accessible via widely used search engines.

### Conclusions

The results of this study suggest that EHIA generated by the for-profit sector is easier to read than EHIA generated by government entities. In order to comply with communication guidelines of the NIH and HHS, government entities and nongovernment organizations would benefit from improving the readability of EHIA.

## Appendix

Multimedia Appendix 1 Individual scores.

Multimedia Appendix 2 Mean (SE) of each organization type.

Multimedia Appendix 3 Pairwise *t* test of Flesch Kincaid Grade.

Multimedia Appendix 4 Pairwise *t* test of SMOG Index.

Multimedia Appendix 5 Pairwise *t* test of Coleman Liau Index.

Multimedia Appendix 6 Pairwise *t* test of Automated Readability Index.

## Abbreviations

e-cigarettes: electronic cigarettes
EHIA: e-cigarette related health information and advice
HHS: US Department of Health and Human Services
NIH: National Institutes of Health
SMOG: Simple Measure of Gobbledygook
