# Supplementary material for: The Readability of Electronic Cigarette Health Information and Advice: A Quantitative Analysis of Web-Based Information
Source: JMIR Public Health Surveill. 2017 Jan 6;3(1):e1. doi: 10.2196/publichealth.6687 (PMC5251168; doi:10.2196/publichealth.6687)
Supplement: Multimedia Appendix 1 [file publichealth_v3i1e1_app1.pdf]

Multimedia Appendix 1 - Individual scores

Organization	Country	Organization Type	Flesch kincaid Grade	SMOG Index	Coleman Liau Index	Automated Readability Index	Average	Original URL	Archived URL	Note
Alaska Department of Health and Social Services Division of Public Health	USA	US government entities	10.9	9.6	15.5	13.7	12.4	http://dhss.alaska.gov/dph/Chronic/Pages/Tobacco/TobaccoFreeAlaska/ecigs.aspx	http://www.webcitation.org/6f1nCLl7R	
American Lung Association	USA	Nongovernment organizations	12.7	11.4	16.0	14.8	13.7	http://www.lung.org/stop-smoking/smoking-facts/e-cigarettes-and-lung-health.html	N/A – we were not able to archive the URL	
Australian Government Department of Health	Australia	Non-US government entities	15.2	13.3	16.8	17.0	15.6	https://www.tga.gov.au/community-qa/electronic-cigarettes	http://www.webcitation.org/6f2CsWOu9	
Board of Equalization - State of California	USA	US government entities	13.9	11.7	16.2	15.1	14.2	http://www.boe.ca.gov/ma/pdf/e-CigReport.pdf	http://www.webcitation.org/6f1fhdMWk	We examined “E-Cigarettes and Health Effects of E-cigarettes” section
Boulder County	USA	US government entities	12.0	11.1	15.3	13.4	13.0	http://www.bouldercounty.org/env/healthyhome/pages/ecigarettes.aspx	N/A – we were not able to archive the URL	
Colorado Department of Public Health and Environme	USA	US government entities	10.7	11.3	14.8	12.9	12.4	https://www.colorado.gov/pacific/sites/default/files/HPF_WIC_e-Cigarette-	http://www.webcitation.org/6f1gTNlXS	

nt								Fact-Sheet.pdf		
Consumer Affair	USA	For-profit entities	11.2	10.2	13.9	11.5	11.7	http://www.consumeraffairs.com/e-cigarettes/#guide-features	http://www.webcitation.org/6f2AmUkHm	We excluded "Expert reviews" section and material consists of advice on using e-cigarette only
Consumer Reports	USA	Nongovernment organizations	11.4	9.9	14.0	14.4	12.4	http://www.consumerreports.org/cro/health/e-cigarette-guide/index.htm	http://www.webcitation.org/6f1rp3gr4	
E Cigarettes Store	N/A	For-profit entities	10.1	8.9	9.4	10.6	9.8	http://www.ecigarettesstore.info/category/advice/	http://www.webcitation.org/6f2Jce2Dt	
Electronic Cigarettes	England	For-profit entities	10.8	10.2	12.5	11.2	11.2	http://www.eelectroniccigarette.co.uk/electronic-cigarettes/smoking-cessation-with-electronic-cigarettes/	http://www.webcitation.org/6f2EHn4O6	
Electronic Cigarettes Reviews	N/A	For-profit entities	9.6	9.3	10.7	10.9	10.1	https://www.electroniccigarettesreviews.net/buying-online	http://www.webcitation.org/6f2ItroUR	Material consists of advice on using e-cigarette only
electroniccigaretteadvice	USA	For-profit entities	7.3	7.5	7.6	7.2	7.4	http://www.electroniccigaretteadvice.org/	N/A – we were not able to archive the URL	

FDA (US Food and Drug Administration) (Consumer Health Information)	USA	US government entities	14.6	12.5	16.7	18.8	15.6	http://www.fda.gov/downloads/ForConsumers/ConsumerUpdates/UCM173430.pdf	http://www.webcitation.org/6f2B8Eo8Q	
FDA (US Food and Drug Administration) (FAQ)	USA	US government entities	13.4	11.7	15.4	15.0	13.9	http://www.fda.gov/ForConsumers/ConsumerUpdates/ucm225210.htm	http://www.webcitation.org/6f1eAJ7Tv	
FDA (US Food and Drug Administration) (General Info)	USA	US government entities	16.1	13.3	17.5	18.5	16.4	http://www.fda.gov/NewsEvents/PublicHealthFocus/ucm172906.htm	http://www.webcitation.org/6f0hYhBfX	
Health Canada	Canada	Non-US government entities	15.8	12.0	16.0	18.3	15.5	http://www.healthycanadians.gc.ca/recall-alert-rappel-avis/hc-sc/2009/13373a-eng.php	http://www.webcitation.org/6f2KM2PtP	
HHS (US Health & Human Services Department)	USA	US government entities	12.6	11.2	16.0	14.8	13.7	http://betobaccofree.hhs.gov/about-tobacco/Electronic-Cigarettes/	http://www.webcitation.org/6f0i2xcor	
King County Public Health	USA	US government entities	13.6	11.4	17.0	15.3	14.3	http://www.kingcounty.gov/healthservices/health/tobacco/tobacco-vapor/e-cigarettes.aspx	http://www.webcitation.org/6f1kCOxp1	
Maine Center for Disease	USA	US government	11.5	10.2	15.4	13.0	12.5	http://www.maine.gov/deh/healthben	http://www.webcitation.org/6f1gtdHZ	

Control and Prevention		entities						es/wellness/documents/13-25-0009E-CigFacts-HlthPro.pdf	V	
Mayo Clinic (FAQ)	USA	Nongovernment organizations	12.8	12.1	16.0	13.8	13.7	http://www.mayoclinic.org/healthy-lifestyle/quit-smoking/expert-answers/electronic-cigarettes/faq-20057776	http://www.webcitation.org/6f1r7Rj1v	
Mayo Clinic (General Info)	USA	Nongovernment organizations	12.0	10.5	16.1	14.0	13.2	http://newsnetwork.mayoclinic.org/discussion/mayo-clinic-experts-what-should-you-know-about-e-cigarettes-26c7a6/	http://www.webcitation.org/6f1rGVd8i	
Michigan Department of Health and Human Services	USA	US government entities	13.9	12.5	16.8	16.8	15.0	https://www.michigan.gov/documents/mdch/E_Cigarette_Facts_465520_7.pdf	http://www.webcitation.org/6f1klrSQU	
Ministry of Health New Zealand	New Zealand	Non-US government entities	12.9	11.8	15.6	14.5	13.7	http://www.health.govt.nz/our-work/preventative-health-wellness/tobacco-control/advice-use-e-cigarettes	http://www.webcitation.org/6f2DS6JMH	
Minnesota department of health	USA	US government entities	12.7	11.3	16.6	14.1	13.7	http://www.health.state.mn.us/ecigarettes	http://www.webcitation.org/6f1nyg1Je	
National Institute on Drug Abuse (NIDA)	USA	US government entities	12.6	10.3	14.4	13.4	12.7	https://teens.drugabuse.gov/drug-facts/tobacco-	N/A – we were not able to archive the URL	We examined “What About E-

		(teen)						nicotine-e-cigarettes		Cigarettes?" section
NC Department of Health and Human Services - Tobacco Prevention and Control Branch	USA	US government entities	13.5	11.2	15.0	14.9	13.6	http://www.tobaccopreventionandcontrol.ncdhhs.gov/ecigs/	http://www.webcitation.org/6f1fFVxw9	
Nebraska Department of Health & Human Services	USA	US government entities	11.6	10.6	13.7	12.9	12.2	http://dhhs.ne.gov/publichealth/Pages/tfn_arr_tfnecig.aspx	http://www.webcitation.org/6f1gZHDRZ	
NHS (National Health Service) UK	UK	Non-US government entities	12.6	11.0	13.3	12.6	12.4	http://www.nhs.uk/smokefree/help-and-advice/e-cigarettes	http://www.webcitation.org/6f2KUVLpr	
NIDA (National Institute on Drug Abuse)	USA	US government entities (teen)	7.3	8.5	10.5	8.5	8.7	http://teens.drugabuse.gov/blog/post/are-e-cigarettes-harmful	http://www.webcitation.org/6f1jrPye3	
NIH (National Institutes of Health): National Institute on Drug Abuse (E-cigarettes and E-hookahs)	USA	US government entities	8.6	8.0	12.1	9.8	9.6	https://www.nlm.nih.gov/medlineplus/ency/patientinstructions/000761.htm	http://www.webcitation.org/6f1jJ3gUn	

NIH (National Institutes of Health): National Institute on Drug Abuse (FAQ)	USA	US government entities	15.3	12.2	16.3	16.7	15.1	https://www.drugabuse.gov/publications/drugfacts/electronic-cigarettes-e-cigarettes http://www.drugabuse.gov/sites/default/files/drugfacts_ecigs.pdf	http://www.webcitation.org/6f1f3IW58	
NIH (National Institutes of Health): National Institute on Drug Abuse (Summary)	USA	US government entities	11.8	10.8	14.9	12.8	12.6	https://www.nlm.nih.gov/medlineplus/ecigarettes.html	http://www.webcitation.org/6f1exxn6e	
North Dakota Department of Health	USA	US government entities	10.7	10.6	15.5	13.4	12.6	https://www.ndhealth.gov/Tobacco/Facts/E-cigs.pdf	http://www.webcitation.org/6f1fKpl8u	
NSW (New South Wales) Government Health	Australia	Non-US government entities	12.9	12.5	14.2	14.9	13.6	http://www.health.nsw.gov.au/tobacco/Pages/electronic-cigarettes.aspx	http://www.webcitation.org/6f2DCv3sg	
Oregon Public Health (General Info)	USA	US government entities	17.0	13.3	16.9	19.2	16.6	http://public.health.oregon.gov/PreventionWellness/TobaccoPrevention/SmokefreeWorkplaceLaw/Pages/e-cigarettes.aspx	http://www.webcitation.org/6f1fYhguF	
Oregon Public Health (News Letter)	USA	US government entities	13.6	11.9	15.6	16.7	14.4	http://public.health.oregon.gov/DiseasesConditions/CommunicableDisease/CDSummaryNewsletter/Documents/2013/ohd6227.pdf	http://www.webcitation.org/6f1njfEDn	

Public Health England	UK	Non-US government entities	13.1	10.6	14.2	16.4	13.6	https://www.gov.uk/government/news/e-cigarettes-around-95-less-harmful-than-tobacco-estimates-landmark-review	N/A – we were not able to archive the URL	
Public Health Law Center	USA	Nongovernment organizations	19.7	14.8	19.2	22.7	19.1	http://publichealthlawcenter.org/topics/tobacco-control/e-cigarettes	http://www.webcitation.org/6f1eieZ1i	
SafeSmoke Australian Laws	Australia	For-profit entities	13.6	10.6	13.1	14.7	13.0	http://safesmoke.com.au/australian-laws/	http://www.webcitation.org/6f2Doz2gM	
SAMHSA (Substance Abuse and Mental Health Services Administration)	USA	US government entities	12.7	11.3	15.0	14.1	13.3	http://www.samhsa.gov/samhsaNewsLetter/Volume_22_Number_3/e_cigarettes/	N/A – we were not able to archive the URL	
South tyneside council	UK	Non-US government entities	18.0	13.3	14.5	21.2	16.7	http://www.southtyneside.gov.uk/article/26329/electronic-cigarette-safety-information	http://www.webcitation.org/6f2CcQ7RD	
Tobacco Control Research Branch of the National Cancer Institute (General Info)	USA	US government entities	9.6	8.2	11.6	10.3	9.9	http://smokefree.gov/E-cigarettes	http://www.webcitation.org/6f1eSZYuP	

Tobacco Control Research Branch of the National Cancer Institute (Teen)	USA	US government entities (teen)	10.2	9.6	12.5	10.6	10.7	http://teen.smokefree.gov/e-cigs.aspx	http://www.webcitation.org/6f1fPbZmF	
U.S. Fire Administration	USA	US government entities	12.3	11.5	16.1	14.5	13.6	https://www.usfa.fema.gov/downloads/pdf/publications/electronic_cigarettes.pdf	http://www.webcitation.org/6f1f8Zz9R	We examined “Health Safety” section
Utah Davis County Health Department	USA	US government entities	11.9	10.8	14.2	12.4	12.3	https://www.daviscountyutah.gov/health/environmental-health-services/permits/businesses/e-cigarettes	http://www.webcitation.org/6f2BOqqYu	
Utah Department of Health	USA	US government entities	12.4	11.1	16.0	14.5	13.5	http://health.utah.gov/tobacco/pdfs/E-Cigarette1.pdf	http://www.webcitation.org/6f2C6TdLw	
Vapegrl	N/A	For-profit entities	12.3	11.3	12.8	13.1	12.4	http://vapegrl.com/e-cigarette-tips-and-tricks/	http://www.webcitation.org/6f2Job1HC	Material consists of advice on using e-cigarette only
Veppo	Australia	For-profit entities	10.6	9.3	12.9	11.2	11.0	http://www.veppocig.com/electronic-cigarettes-with-nicotine-for-australia/	N/A – we were not able to archive the URL	
Vermont Department of Health	USA	US government entities	14.6	12.4	18.7	17.8	15.9	http://healthvermont.gov/prevent/tobacco/documents/Ecigarette_factsheet.p	http://www.webcitation.org/6f1ms6xAf	

								df		
Washington State Department of Health	USA	US government entities	10.3	9.9	14.0	11.9	11.5	http://www.doh.wa.gov/YouandYourFamily/Tobacco/OtherTobaccoProducts/ECigarettes	http://www.webcitation.org/6f1hIUZSM	
WebMD	USA	For-profit entities	7.9	8.1	12.4	9.3	9.4	http://www.webmd.com/smoking-cessation/features/electronic-cigarettes	http://www.webcitation.org/6f1rbWmFX	
White Smoke Review	N/A	For-profit entities	8.0	8.3	9.8	8.6	8.7	http://whitesmokereview.com/e-cigarette-tips/	http://www.webcitation.org/6f2JCQqXY	Material consists of advice on using e-cigarette only
WHO (World Health Organization)	United Nations	Non-Government Organizations	12.7	10.7	14.0	14.5	13.0	http://www.who.int/bulletin/volumes/92/12/14-031214/en/ http://www.who.int/bulletin/volumes/92/12/14-031214.pdf http://dx.doi.org/10.2471/BLT.14.031214	http://www.webcitation.org/6f1ec5d0u	
Wikimedia Foundation	USA	Non-Government Organizations	14.2	12.2	16.7	17.1	15.1	https://en.wikipedia.org/wiki/Electronic_cigarette	http://www.webcitation.org/6f1pGP9NL	We examined “Health Effects” section
