# Supplementary material for: The Readability of Electronic Cigarette Health Information and Advice: A Quantitative Analysis of Web-Based Information
Source: JMIR Public Health Surveill. 2017 Jan 6;3(1):e1. doi: 10.2196/publichealth.6687 (PMC5251168; doi:10.2196/publichealth.6687)
Supplement: Multimedia Appendix 2 [file publichealth_v3i1e1_app2.pdf]

Multimedia Appendix 2 - Mean (SE) of each organization type

Organization Type	Freq.	Mean (St. Err.) of Flesch kincaid Grade	Mean (St. Err.) of Smog Index	Mean (St. Err.) of Coleman Liau Index	Mean (St. Err.) of Automated Readability Index	Mean (St. Err.) of 4 metrics
For-profit entities	10	10.14 (0.64)	9.37 (0.38)	11.52 (0.64)	10.83 (0.68)	10.46 (0.55)
Nongovernment organizations	7	13.64 (1.06)	11.66 (0.61)	16.00 (0.67)	15.90 (1.21)	14.30 (0.86)
Non-US government entities	7	14.36 (0.77)	12.07 (0.40)	14.93 (0.46)	16.41 (1.06)	14.44 (0.58)
US government	27	12.66 (0.37)	11.17 (0.24)	15.51 (0.29)	14.57 (0.46)	13.48 (0.33)
US government entities written for teens	3	10.03 (1.53)	9.47 (0.52)	12.50 (1.13)	10.83 (1.42)	10.71 (1.15)
