# Supplementary material for: The Readability of Electronic Cigarette Health Information and Advice: A Quantitative Analysis of Web-Based Information
Source: JMIR Public Health Surveill. 2017 Jan 6;3(1):e1. doi: 10.2196/publichealth.6687 (PMC5251168; doi:10.2196/publichealth.6687)
Supplement: Multimedia Appendix 3 [file publichealth_v3i1e1_app3.pdf]

Multimedia Appendix 3 - Pairwise *t* test of Flesch Kincaid Grade

Organization Type	Organization Type	***t* value**	***P* value**	**Adjusted p-value (Hommel)**
Versus for-profit entities	Nongovernment organizations	-3.01	.009	.07
	Non-US government entities	-4.23	.001	.007
	US government	-3.49	.001	.01
	US government (teen)	0.08	.94	.94
Versus nongovernment organizations	Non-US government entities	-0.55	.60	.94
	US government	1.09	.28	.85
	US government (teen)	1.89	.10	.38
Versus non-US government entities	US government	2.04	.05	.24
	US government (teen)	2.84	.02	.13
Versus US government	US government (teen)	2.16	.04	.20
