# Supplementary material for: The Readability of Electronic Cigarette Health Information and Advice: A Quantitative Analysis of Web-Based Information
Source: JMIR Public Health Surveill. 2017 Jan 6;3(1):e1. doi: 10.2196/publichealth.6687 (PMC5251168; doi:10.2196/publichealth.6687)
Supplement: Multimedia Appendix 4 [file publichealth_v3i1e1_app4.pdf]

Multimedia Appendix 4 - Pairwise *t* test of SMOG Index

Organization Type	Organization Type	***t* value**	***P* value**	**Adjusted p-value (Hommel)**
Versus for-profit entities	Nongovernment organizations	-3.35	.004	.03
	Non-US government entities	-4.78	<.001	.002
	US government	-3.90	<.001	.004
	US government (teen)	-0.13	.90	.90
Versus nongovernment organizations	Non-US government entities	-0.57	.58	.90
	US government	0.86	.40	.87
	US government (teen)	2.15	.06	.26
Versus non-US government entities	US government	1.74	.09	.37
	US government (teen)	3.72	.006	.04
Versus US government	US government (teen)	2.26	.03	.18
