# Supplementary material for: The Readability of Electronic Cigarette Health Information and Advice: A Quantitative Analysis of Web-Based Information
Source: JMIR Public Health Surveill. 2017 Jan 6;3(1):e1. doi: 10.2196/publichealth.6687 (PMC5251168; doi:10.2196/publichealth.6687)
Supplement: Multimedia Appendix 5 [file publichealth_v3i1e1_app5.pdf]

Multimedia Appendix 5 - Pairwise *t* test of Coleman Liau Index

Organization Type	Organization Type	***t* value**	***P* value**	**Adjusted p-value (Hommel)**
Versus for-profit entities	Nongovernment organizations	-4.71	<.001	.003
	Non-US government entities	-3.94	.001	.01
	US government	-6.45	<.001	<.001
	US government (teen)	-0.74	.48	.48
Versus nongovernment organizations	Non-US government entities	1.32	.21	.48
	US government	0.74	.47	.48
	US government (teen)	2.80	.02	.12
Versus non-US government entities	US government	-0.93	.36	.48
	US government (teen)	2.45	.04	.20
Versus US government	US government (teen)	3.17	.004	.03
