# Supplementary material for: The Readability of Electronic Cigarette Health Information and Advice: A Quantitative Analysis of Web-Based Information
Source: JMIR Public Health Surveill. 2017 Jan 6;3(1):e1. doi: 10.2196/publichealth.6687 (PMC5251168; doi:10.2196/publichealth.6687)
Supplement: Multimedia Appendix 6 [file publichealth_v3i1e1_app6.pdf]

**Multimedia Appendix 6 - Pairwise *t* test of Automated Readability Index**

| Organization Type | Organization Type | ***t* value** | ***P* value** | **Adjusted p-value (Hommel)** |
|---|---|---|---|---|
| Versus for-profit entities | Nongovernment organizations | -3.94 | .001 | .01 |
| | Non-US government entities | -4.66 | <.001 | .003 |
| | US government | -4.34 | <.001 | .001 |
| | US government (teen) | -0.002 | 1.00 | 1.00 |
| Versus nongovernment organizations | Non-US government entities | -0.32 | .75 | 1.00 |
| | US government | 1.23 | .23 | .69 |
| | US government (teen) | 2.43 | .04 | .21 |
| Versus non-US government entities | US government | 1.76 | .09 | .35 |
| | US government (teen) | 2.97 | .02 | .11 |
| Versus US government | US government (teen) | 2.56 | .02 | .10 |
